# Profound Hypothyroidism as a Reversible Cause of Severe Low-Density Lipoprotein Cholesterol (LDL-C) Elevation: A Case Report

**DOI:** 10.7759/cureus.113365

**Published:** 2026-07-25

**Authors:** Nirosh E Mataraarachchi, Shalini Bhat

**Affiliations:** 1 Medicine, University of California, Los Angeles, Los Angeles, USA; 2 Endocrinology, Diabetes and Metabolism, University of California, Los Angeles, Los Angeles, USA; 3 Endocrinology, Diabetes and Metabolism, VA Greater Los Angeles Healthcare System, Los Angeles, USA

**Keywords:** apolipoprotein b, familial hypercholesterolemia, hashimoto thyroiditis, hypothyroidism, reversible hyperlipidemia, secondary dyslipidemia, severe hypercholesterolemia

## Abstract

Thyroid hormone plays a central role in regulating lipid metabolism. In states of profound hypothyroidism, impaired triiodothyronine (T3)-mediated regulation of hepatic LDL (low-density lipoprotein) receptor expression results in decreased clearance of apolipoprotein B-containing lipoproteins. These physiologic disturbances can lead to substantial low-density lipoprotein cholesterol (LDL-C) elevation, which can mimic primary genetic lipid disorders and complicate the clinical evaluation of severe hypercholesterolemia.

Herein, we describe a patient with autoimmune thyroid disease status post total thyroidectomy who demonstrated marked, reproducible LDL-C elevations during periods of profound hypothyroidism, with substantial improvement upon restoration of euthyroidism. These fluctuations occurred in the absence of lipid-lowering therapy.

This case highlights the importance of assessing thyroid function in patients with severe LDL-C elevation. Recognition and treatment of hypothyroidism can substantially improve lipid abnormalities and reveal a reversible endocrine-mediated contributor to marked hypercholesterolemia.

## Introduction

Hypothyroidism is a well-established secondary cause of dyslipidemia [1,2]. Thyroid hormone regulates lipid metabolism through effects on hepatic lipoprotein clearance, and abnormalities in thyroid function are frequently associated with alterations in serum lipid profiles [2,3]. Hypothyroidism is associated with elevations in both total cholesterol and low-density lipoprotein cholesterol (LDL-C), primarily due to impaired clearance of circulating apolipoprotein B (ApoB)-containing lipoproteins [2]. Mild thyroid hormone deficiency can lead to measurable changes in lipid parameters, while more severe hypothyroidism may result in substantial hypercholesterolemia [2,4]. In profound hypothyroidism, LDL-C elevations may approach levels typically associated with familial hypercholesterolemia (FH), i.e., persistently exceeding approximately 190 mg/dL in adults per diagnostic frameworks such as the Dutch Lipid Clinic Network and Simon Broome criteria [5]. Recognition of thyroid dysfunction is clinically important.

This case highlights a striking longitudinal relationship between profound hypothyroidism and marked LDL-C elevation in a patient with autoimmune thyroid disease following thyroidectomy. The objective of this report is to emphasize the importance of evaluating thyroid function in patients presenting with severe hypercholesterolemia and to illustrate the reversible nature of hypothyroidism-associated lipid abnormalities with appropriate endocrine management.

## Case presentation

**Figure 1:**
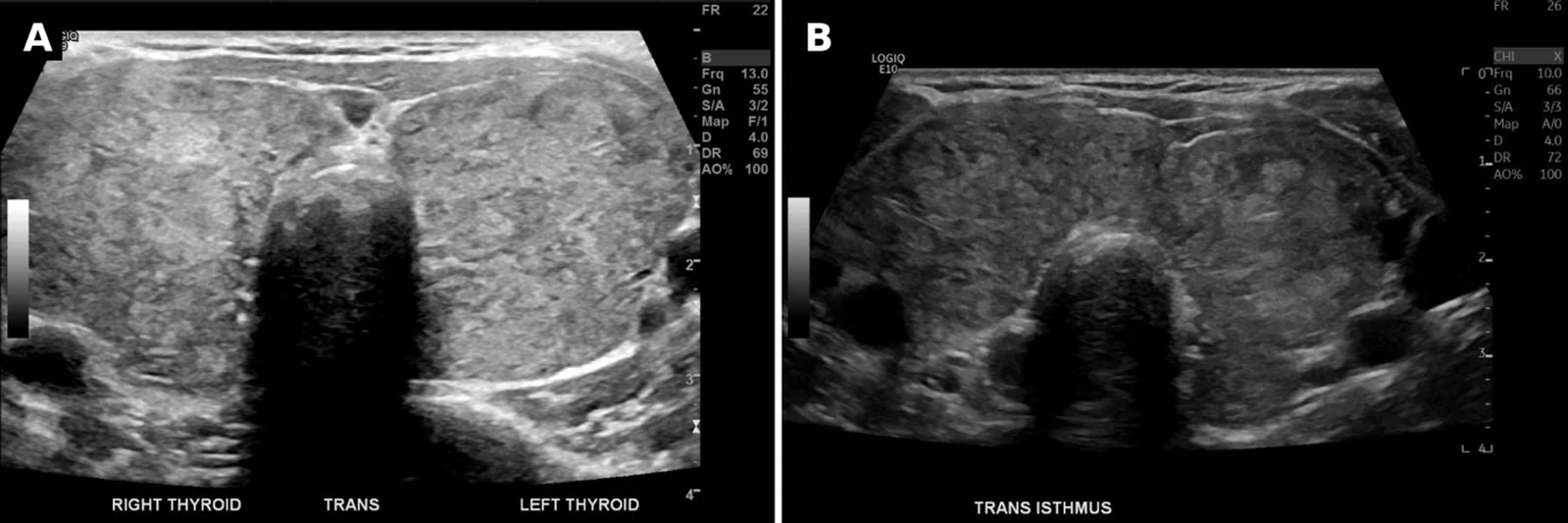
Serial thyroid imaging demonstrating progressive gland enlargement over time (A) Transverse ultrasound, Year 1 (12/2018): diffusely enlarged, heterogeneous thyroid gland without evidence of a discrete nodule (right lobe 5.2 × 2.7 × 2.4 cm; left lobe 4.9 × 2.7 × 2.4 cm). (B) Transverse ultrasound, Year 3 (10/2020): markedly heterogeneous and hypervascular gland compatible with thyroiditis, with a new 1.4 × 1.1 × 1.1 cm hyperechoic right lobe nodule (right lobe 7.0 × 3.0 × 2.7 cm; left lobe 7.1 × 4.3 × 2.6 cm).

A 47-year-old woman was incidentally found to have thyroid enlargement in 2018 (Year 1). Thyroid ultrasound demonstrated a diffusely enlarged, heterogeneous gland without discrete nodules, measuring 5.2 × 2.7 × 2.4 cm (right lobe) and 4.9 × 2.7 × 2.4 cm (left lobe) at initial presentation in Year 1 (Figure 1). Thyroid function tests were normal, while thyroid peroxidase and anti-thyroglobulin antibodies were markedly elevated, consistent with Hashimoto thyroiditis.

**Figure 2:**
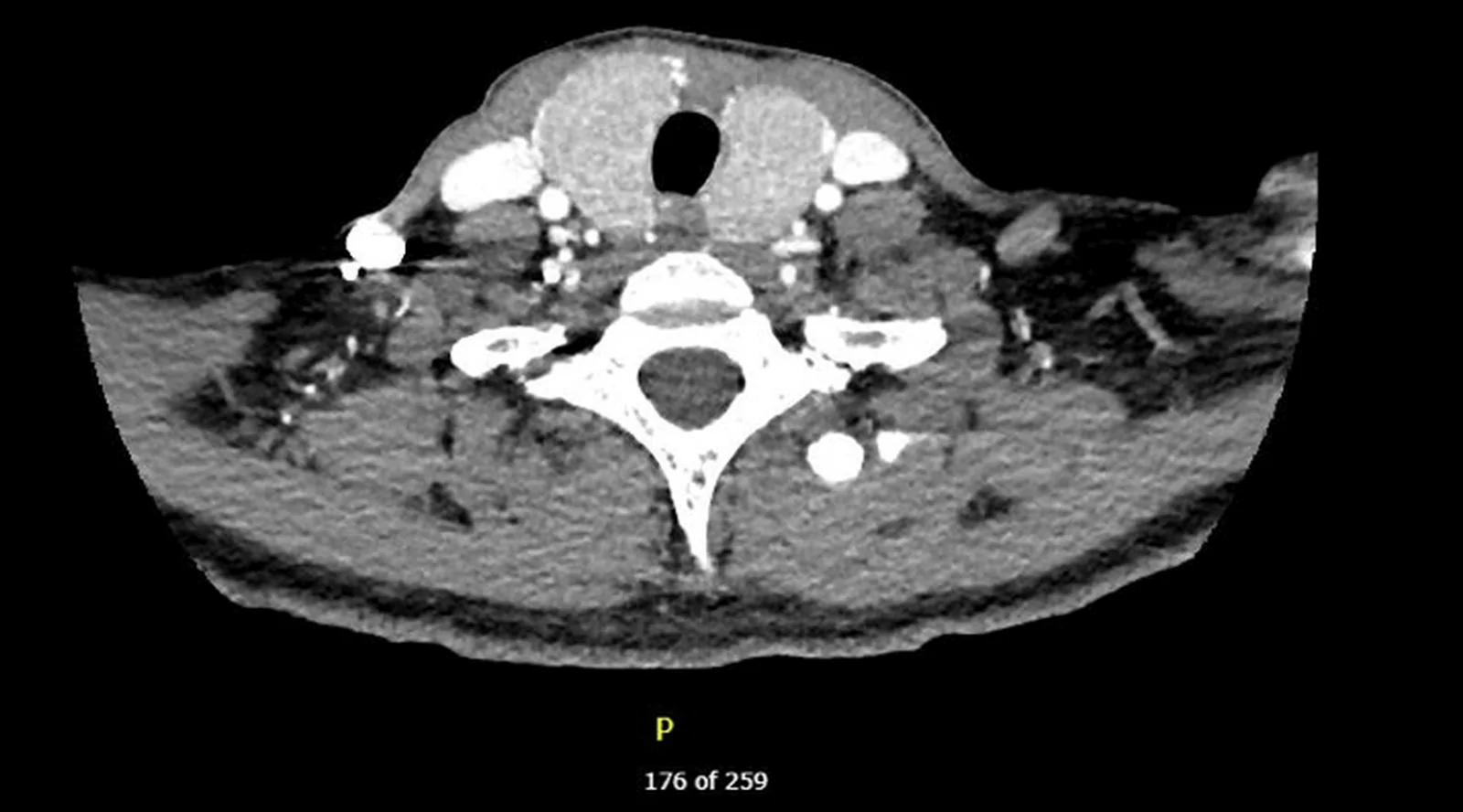
Axial contrast-enhanced CT, Year 4 (1/2021) Diffusely enlarged thyroid gland with multiple nodules, including a subcentimeter enhancing nodule in the right upper pole, and an isthmus thickness of 1.7 cm, extending from the hyoid bone to the superior border of the clavicles (right lobe 6.7 × 4.0 × 2.5 cm; left lobe 7.0 × 4.0 × 2.4 cm).

Over subsequent years, serial imaging demonstrated progressive thyroid enlargement, accompanied by dysphagia and neck fullness. Thyroid lobe dimensions increased to 7.0 × 3.0 × 2.7 cm (right lobe) and 7.1 × 4.3 × 2.6 cm (left lobe) in Year 3, with a new 1.4 × 1.1 × 1.1 cm hyperechoic right lobe nodule, then to 6.7 × 4.0 × 2.5 cm (right lobe) and 7.0 × 4.0 × 2.4 cm (left lobe) in Year 4 (Figures 1, 2). In the setting of persistent compressive symptoms and continued thyroid enlargement, she underwent total thyroidectomy in Year 5. Surgical pathology confirmed Hashimoto thyroiditis without malignancy.

Postoperatively, the medication burden and need to separate calcium from levothyroxine complicated long-term thyroid hormone replacement. The patient reported intermittent difficulty maintaining the regimen.

A striking, reproducible biochemical pattern emerged in which the patient's LDL-C levels rose and fell in parallel with the degree of hypothyroidism. During periods of profound hypothyroidism, thyroid-stimulating hormone (TSH) levels reached 228.4 mIU/L (reference range, 0.55-4.78 mIU/L) with free thyroxine (FT4) as low as 0.10-0.31 ng/dL (reference range, 0.8-1.8 ng/dL), accompanied by marked hypercholesterolemia. In November 2023 (Year 6), TSH was 42.2 mIU/L with total cholesterol 291 mg/dL (reference range, <200 mg/dL) and LDL-C 205 mg/dL (reference range, <100 mg/dL). With improved levothyroxine adherence, thyroid function normalized by July 2024 (Year 7) (TSH 1.8 mIU/L), and LDL-C fell to 110 mg/dL. Severe hypothyroidism recurred in January 2026 (Year 9) (TSH 173.2 mIU/L) with LDL-C peaking at 259 mg/dL and total cholesterol 367 mg/dL; ApoB was elevated at 139 mg/dL (reference range, <90 mg/dL), whereas lipoprotein(a) (Lp(a)) was not elevated at 73 nmol/L (reference range, <75 nmol/L), and triglycerides remained relatively stable (reference range, <150 mg/dL).

**Figure 3:**
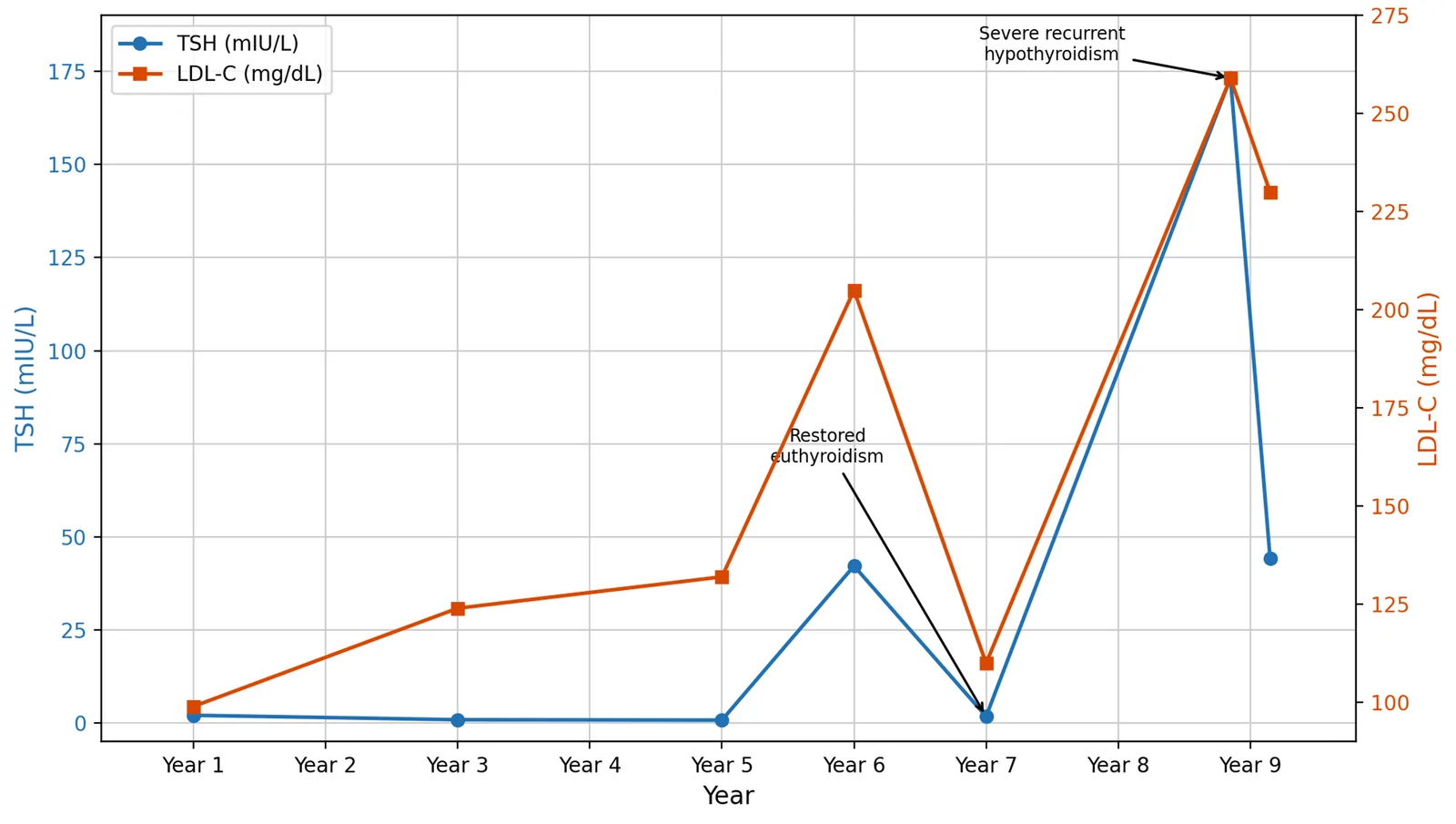
Longitudinal changes in TSH and LDL-C over time. Longitudinal changes in TSH and LDL-C by year elapsed since initial presentation. LDL-C increased during periods of overt hypothyroidism, improved with restoration of euthyroidism, and rose again with recurrent severe hypothyroidism, supporting a reversible thyroid-mediated contribution to marked LDL-C elevation. LDL-C: low-density lipoprotein cholesterol; TSH: thyroid-stimulating hormone

Evaluation for other common secondary causes of hyperlipidemia showed no diabetes mellitus, chronic kidney disease, liver disease, or biochemical evidence of cholestasis. There were no documented tendon xanthomas, xanthelasma, or corneal arcus on examination. Family history was negative for premature atherosclerotic cardiovascular disease (ASCVD) or known familial hypercholesterolemia (FH). Medication review showed no prior or active lipid-lowering therapy during the observation period. Statin therapy had been recommended, but the patient declined because of concern about additional pill burden amid polypharmacy. The longitudinal relationship between TSH and LDL-C is summarized in Table 1 and illustrated in Figure 3.

**Table 1:** Longitudinal Relationship Between Thyroid Function and LDL-C Note: ApoB was elevated at 139 mg/dL during recurrent hypothyroidism in Year 9, supporting increased circulating atherogenic lipoprotein particle burden. Reference ranges: TSH 0.55–4.78 mIU/L and LDL-C <130 mg/dL. No lipid-lowering therapy was prescribed or documented during the period of observation. LDL-C: low-density lipoprotein cholesterol; TSH: thyroid-stimulating hormone

Date	Clinical phase	TSH (mIU/L)	LDL-C (mg/dL)	Interpretation
Year 1	Pre-thyroidectomy, euthyroid baseline	2.1	99	Baseline LDL-C before thyroidectomy
Year 3	Pre-thyroidectomy, euthyroid	0.9	124	LDL-C remained mildly elevated but below severe range
Year 5	Post-thyroidectomy, euthyroid	0.8	132	LDL-C mildly elevated after thyroidectomy
Year 6	Post-thyroidectomy, hypothyroid recurrence	42.2	205	LDL-C increased with worsening hypothyroidism
Year 7	Post-thyroidectomy, restored euthyroidism	1.8	110	LDL-C improved with improved levothyroxine adherence
Year 9 (January)	Post-thyroidectomy, severe recurrent hypothyroidism	173.2	259	Marked LDL-C elevation during severe hypothyroidism
Year 9 (February)	Post-thyroidectomy, persistent overt hypothyroidism	44.2	230	LDL-C remained elevated as hypothyroidism persisted

## Discussion

Severe hypercholesterolemia is a well-recognized but often underappreciated manifestation of overt hypothyroidism [2]. Thyroid hormone regulates lipid metabolism through triiodothyronine (T3)-mediated activation of nuclear thyroid hormone receptors, which control expression of genes involved in cholesterol homeostasis [3,6]. At a molecular level, T3 stimulates LDL receptor gene expression in hepatocytes through activation of the sterol regulatory element-binding protein-2 (SREBP-2) pathway, thereby promoting hepatic uptake and clearance of LDL particles [7]. In states of thyroid hormone deficiency, reduced LDL receptor expression results in decreased LDL clearance and accumulation of circulating ApoB-containing lipoproteins [2,3].

In addition to reduced LDL receptor expression, elevated TSH has been shown to upregulate hepatic proprotein convertase subtilisin/kexin type 9 (PCSK9) expression [8]. Because PCSK9 normally binds LDL receptors and targets them for lysosomal degradation, this elevated expression accelerates LDL receptor turnover. Together, reduced LDL receptor expression and increased PCSK9-mediated LDL receptor degradation create a complementary "dual-hit" mechanism (reduced LDL receptor expression and increased PCSK9-mediated receptor degradation) that may contribute to severe LDL-C elevation in profound hypothyroidism.

Beyond quantitative increases in LDL-C, hypothyroidism has also been associated with increased oxidation of LDL, a key driver of endothelial dysfunction and atherogenesis, which reverses with restoration of euthyroidism [9]. Thus, prolonged hypothyroidism-induced hypercholesterolemia represents not only an elevated LDL burden but also an enhanced atherogenic milieu.

Although hypothyroidism can also impair triglyceride clearance through decreased lipoprotein lipase activity, the effect on triglycerides is variable and often modest compared with the more pronounced LDL-C elevation. In this case, triglyceride levels remained relatively stable despite marked LDL-C elevation, consistent with the predominantly receptor-mediated mechanism of LDL accumulation described above. Hypothyroidism is one of the most common causes of secondary dyslipidemia, with both overt and subclinical disease associated with increased LDL-C [1,2]. The 2020 Endocrine Society Clinical Practice Guideline recommends ruling out hypothyroidism as a cause of hyperlipidemia before initiating lipid-lowering medications [10]. Although dyslipidemia in hypothyroidism is well recognized, severe LDL-C elevations approaching levels typically associated with FH are less commonly described [2]. Failure to recognize hypothyroidism as an underlying cause of marked hypercholesterolemia may delay appropriate treatment and expose patients to prolonged cardiovascular risk.

This distinction is particularly important in patients with markedly elevated LDL-C, in whom primary genetic disorders such as FH must be considered. Because thyroid hormone exerts many of its lipid-lowering effects through upregulation of hepatic LDL receptors, profound hypothyroidism may mimic receptor-mediated forms of hypercholesterolemia, further complicating the diagnosis. FH is classically characterized by a strong family history of premature coronary artery disease, tendon xanthomas, corneal arcus before age 45 years, and persistently elevated LDL-C despite euthyroidism. Clinically, primary FH is suspected when untreated LDL-C persistently exceeds approximately 190 mg/dL in adults, a threshold incorporated into major diagnostic frameworks such as the Dutch Lipid Clinic Network and Simon Broome criteria [5]. In this patient, dynamic LDL-C changes with thyroid status, absence of lipid-lowering therapy, and lack of clinical or family history features suggestive of FH supported severe hypothyroidism as the major driver of hypercholesterolemia. Key clinical and biochemical differences between FH and hypothyroidism-induced hypercholesterolemia are summarized in Table 2.

**Table 2:** Clinical and Biochemical Features Distinguishing Heterozygous Familial Hypercholesterolemia From Hypothyroidism-Induced Hypercholesterolemia FH: familial hypercholesterolemia; LDL-C: low-density lipoprotein cholesterol; TSH: thyroid-stimulating hormone. Table content derived from [1,2,4,6,10] and reflects a literature-based synthesis of established clinical distinctions rather than a validated diagnostic instrument specific to this case.

Feature	Heterozygous FH	Hypothyroidism-Induced Hypercholesterolemia
LDL-C pattern	Persistent elevation, often beginning in childhood	Dynamic; fluctuates with thyroid status
TSH level	Typically normal unless coexisting thyroid disease	Elevated, sometimes profoundly
Response to levothyroxine	No meaningful LDL-C improvement unless hypothyroidism coexists	LDL-C improves with restoration of euthyroidism
Mechanism	Primary disorder of LDL receptor-mediated clearance	Secondary reduction in LDL receptor expression and impaired LDL clearance
Triglycerides	Often normal	Normal or mildly elevated; variable
Physical findings	Tendon xanthomas or corneal arcus may be present	Findings of hypothyroidism may be present; xanthomas and premature corneal arcus are typically absent
Family history	May show autosomal dominant pattern or premature coronary artery disease	No specific familial pattern of severe hypercholesterolemia
Temporal pattern	Lifelong and persistent	Biochemically dynamic; changes with TSH and thyroid hormone adherence
Key diagnostic consideration	Consider FH when LDL-C remains markedly elevated despite euthyroidism	Evaluate thyroid function before diagnosing primary hypercholesterolemia

In our patient, elevated ApoB supported an increased burden of circulating atherogenic lipoprotein particles, consistent with impaired LDL receptor-mediated clearance in severe hypothyroidism. Thyroid hormone replacement led to substantial improvement in the lipid profile, paralleling the restoration of euthyroidism. A systematic review and meta-analysis demonstrated that levothyroxine therapy in overt hypothyroidism is associated with significant decreases in total cholesterol (−58.4 mg/dL), LDL-C (−41.11 mg/dL), and ApoB (−33.96 mg/dL) [4]. This case demonstrates a clear longitudinal relationship between thyroid dysfunction and lipid abnormalities and underscores the importance of identifying hypothyroidism prior to initiating lipid-lowering therapy.

Limitations

This case report has several limitations. Potential confounders, including body weight changes, use of other medications, dietary intake, and intercurrent acute illness, were not systematically assessed at each timepoint and cannot be fully excluded as contributors to the observed lipid fluctuations. Fasting status of the lipid panels was not consistently documented across timepoints, which may affect precise interpretation of LDL-C values, although the effect of fasting state on LDL-C is generally modest. ApoB was measured at a single time point (Year 9) rather than longitudinally, limiting assessment of its correlation with LDL-C and TSH over the course of the patient's disease.

## Conclusions

Profound overt hypothyroidism can result in marked LDL-C elevations that may mimic FH. The underlying mechanism involves a complementary dual-hit process in which thyroid hormone deficiency simultaneously reduces hepatic LDL receptor expression and accelerates receptor degradation, resulting in markedly impaired LDL clearance. Beyond quantitative LDL-C elevation, hypothyroidism also promotes LDL oxidation, amplifying the atherogenic burden during periods of thyroid hormone deficiency. This case illustrates that the degree of LDL-C elevation in profound hypothyroidism can approach levels typically associated with familial hypercholesterolemia, underscoring the importance of thyroid function evaluation before attributing severe hypercholesterolemia to a primary genetic etiology or initiating lipid-lowering therapy. TSH testing should be considered early in the workup of unexplained, marked LDL-C elevation, particularly in patients with known autoimmune thyroid disease, prior thyroid surgery, or risk factors for nonadherence to thyroid hormone replacement. Restoration of euthyroidism through optimized levothyroxine therapy can substantially reverse hypothyroidism-associated lipid abnormalities, potentially obviating the need for pharmacologic lipid-lowering treatment and reducing cardiovascular risk.
